# Benzalkonium chloride-induced myofibroblastic transdifferentiation of Tenon’s capsule fibroblasts is inhibited by coculture with corneal epithelial cells or by interleukin-10

**DOI:** 10.1038/s41598-021-94852-8

**Published:** 2021-08-09

**Authors:** Chiemi Yamashiro, Kazuhiro Tokuda, Yuka Kobayashi, Fumiaki Higashijima, Takuya Yoshimoto, Manami Ota, Tadahiko Ogata, Atsushige Ashimori, Masaaki Kobayashi, Makoto Hatano, Sho-Hei Uchi, Makiko Wakuta, Shinichiro Teranishi, Kazuhiro Kimura

**Affiliations:** Department of Ophthalmology, Yamaguchi University Graduate School of Medicine, Ube, Yamaguchi 755-8505 Japan

**Keywords:** Molecular medicine, Eye diseases

## Abstract

Benzalkonium chloride (BAC) is used as a preservative in eyedrops but induces subconjunctival fibrosis that can result in failure of glaucoma surgery. Tenon’s capsule fibroblasts in subconjunctival tissue interact with the corneal epithelium through tear fluid. With the use of a coculture system, we have now investigated the effect of human corneal epithelial (HCE) cells on myofibroblastic transdifferentiation of human Tenon fibroblasts (HTFs) induced by BAC (5 × 10^−6^%). Immunofluorescence and immunoblot analyses revealed that the BAC-induced expression of α smooth muscle actin (αSMA) in HTFs was suppressed by coculture of these cells with HCE cells (*p* < 0.01). The concentration of interleukin-10 (IL-10) in culture supernatants of BAC-treated HTFs was increased by coculture with HCE cells (17.26-fold, vs. coculure, *p* < 0.001). Immunofluorescence and immunoblot analyses also showed that exogenous IL-10 (300 pg/ml) suppressed the BAC-induced expression of αSMA by 43.65% (*p* < 0.05) as well as the nuclear translocation of myocardin-related transcription factor-A (MRTF-A) by 39.32% (*p* < 0.01) in HTFs cultured alone. Our findings suggest that corneal epithelial cells may protect against subconjunctival fibrosis by maintaining IL-10 levels and preventing the MRTF-A-dependent transdifferentiation of HTFs into myofibroblasts.

## Introduction

Glaucoma is characterized by a gradual narrowing of the visual field that eventually leads to blindness. One of the most important risk factors for glaucoma is high intraocular pressure. Treatment with eyedrops or filtration surgery to lower intraocular pressure is necessary in order to attenuate the progressive vision loss in individuals with glaucoma. Most antiglaucoma eyedrops contain a preservative to prevent the growth of microorganisms. Benzalkonium chloride (BAC) is the most widely used preservative for eyedrops^1^. However, exposure to BAC during the treatment of glaucoma with eyedrops can have harmful effects on the ocular surface, including the induction of ocular discomfort, tear film instability, conjunctival inflammation, and damage to the corneal and conjunctival epithelium^1–4^. Glaucoma filtration surgery involves the formation of a filtering bleb composed of subconjunctival tissue that contributes to a sustained lowering of intraocular pressure. The failure rate for glaucoma filtration surgery is also increased by long-term use of glaucoma eyedrops containing BAC before surgery^5,6^, an effect that is thought to reflect the induction of subconjunctival fibrosis by BAC^1,3,7,8^. Maintenance of the filtering bleb after surgery remains a challenge in the glaucoma clinic.

Tissue-resident fibroblasts, as well as circulating progenitors, epithelial cells, and endothelial cells, have the potential to transdifferentiate into myofibroblasts and thereby to contribute to fibrosis^9,10^. Myofibroblasts are characterized by the overexpression of α smooth muscle actin (αSMA), the formation of actin stress fibers, and the overproduction of extracellular matrix (ECM) proteins such as collagen types I and III and glycoproteins in response to various stimuli including injury, infection, inflammation, and stress. Myocardin-related transcription factor (MRTF) signaling plays an important role in the differentiation and activation of myofibroblasts^11,12^. We have previously shown that MRTF signaling is associated with epithelial-mesenchymal transition (EMT) of retinal pigment epithelial cells^13^. MRTF-A binds to the promoter region and thereby regulates the expression of myofibroblast-related genes such as those for αSMA, fibronectin, and connective tissue growth factor (CTGF)^14^. The immune system contributes to the regulation of myofibroblasts^15^, and the anti-inflammatory cytokine interleukin (IL)-10 is a potential therapeutic target for modulation of tissue remodeling^10^.

Histological specimens of failed filtration structures after glaucoma surgery show an increased number of αSMA-positive cells and high-density deposition of collagen types I or III compared with corresponding normal tissue^16,17^. BAC was shown to shorten bleb survival time after rabbit glaucoma filtration surgery as well as to increase the expression of αSMA in the filtration bleb^18^.

The cornea, conjunctiva, and eyelids maintain ocular surface homeostasis and interact in pathological changes that affect each tissue^19^. The corneal epithelium plays an important role as a barrier at the surface of the eye. Disruption of this barrier allows the spread of inflammation from the conjunctiva or eyelid into the cornea^19^. The corneal epithelium and subconjunctival tissue also communicate with each other via tear fluid^19^. However, the possible effect of the corneal epithelium on the BAC-induced transdifferentiation of Tenon’s capsule fibroblasts into myofibroblasts has been unknown.

To address this question, we have now examined the effect of BAC on the transdifferentiation of human Tenon’s capsule fibroblasts (HTFs) into myofibroblasts in a coculture system with simian virus 40 (SV40)-immortalized human corneal epithelial (HCE) cells. We also examined the possible protective effect of IL-10 released into the coculture supernatant on the observed deleterious action of BAC on HTFs in this system.

## Results

### Cytotoxicity of BAC for HTFs and HCE cells

**Figure 1:**
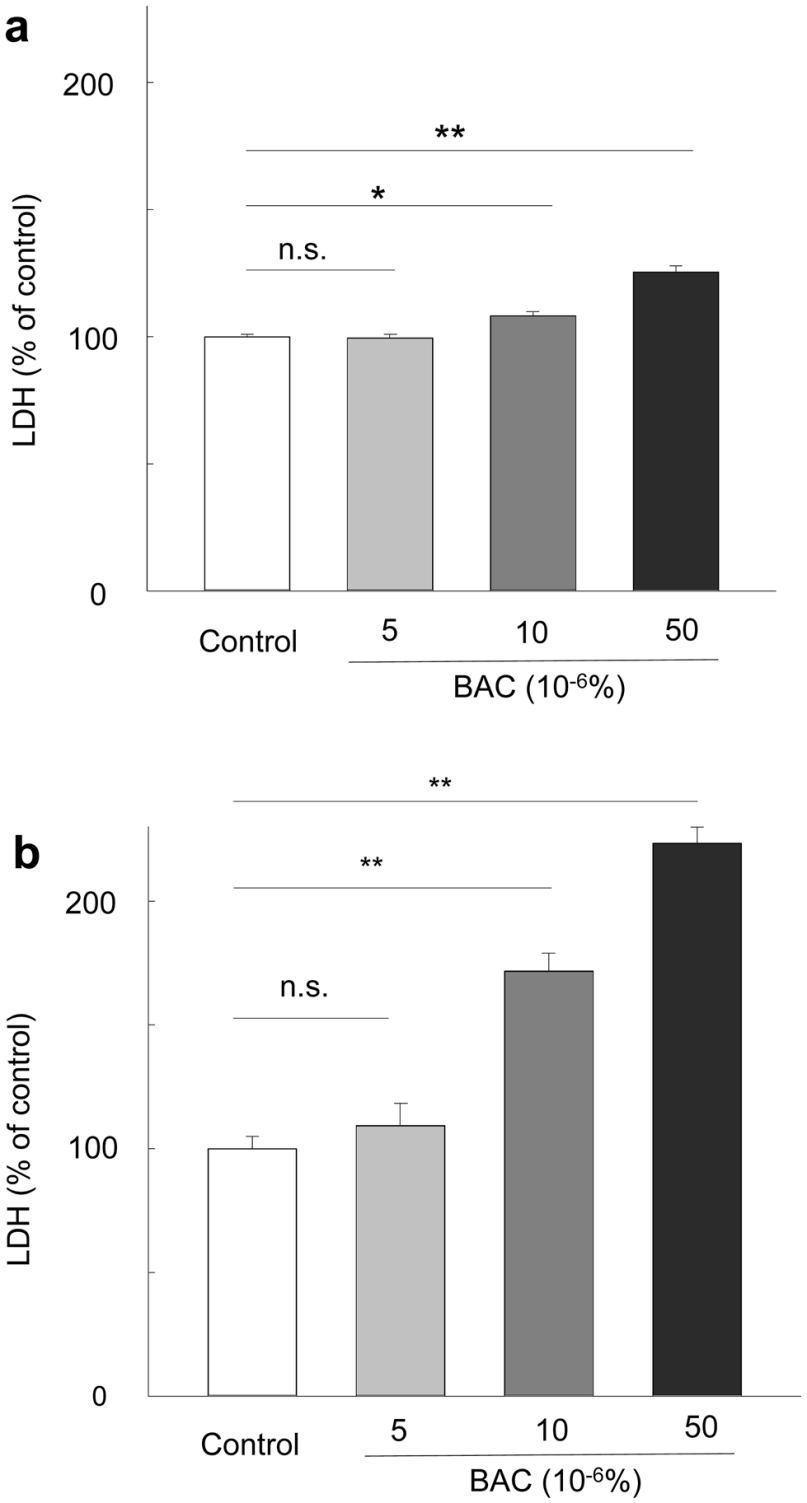
Cytotoxicity of BAC for HTFs and HCE cells. HTFs (**a**) or HCE cells (**b**) were deprived of serum for 24 h and then incubated in the absence (Control) or presence of the indicated concentrations of BAC for 24 h, after which the amount of LDH released into the medium was measured. Data are expressed as a percentage of the control value and are means + s.e.m. of five independent experiments. *p < 0.05, **p < 0.01; n.s., not significant (Holm-Sidak post hoc test).

We first examined the effect of BAC on the viability of HTFs and HCE cells by measuring the release of lactate dehydrogenase (LDH) into the culture medium. The cells were deprived of serum for 24 h and then exposed to various concentrations of BAC for 24 h. We found that the amount of LDH in the medium of HTFs (*p* = 0.014) or HCE (*p* < 0.001) cells was increased by exposure to BAC in a concentration-dependent manner, with this effect being significant at BAC concentrations of ≥ 10 × 10^−6^% (Fig. 1). We therefore performed subsequent experiments with BAC at the nontoxic concentration of 5 × 10^−6^%.

### Effect of BAC on HTF transdifferentiation into myofibroblasts

**Figure 2:**
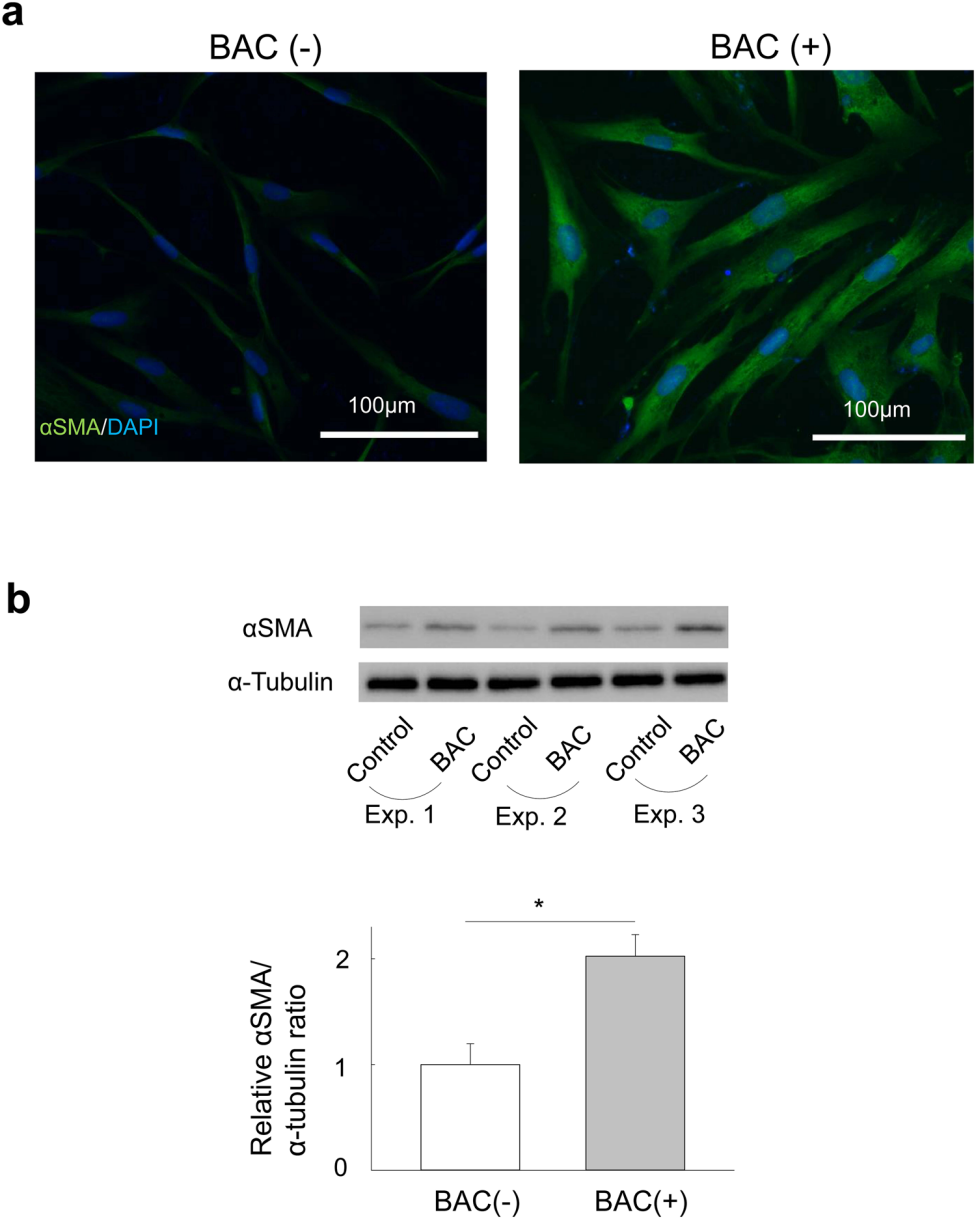
Effect of BAC on the transdifferentiation of HTFs into myofibroblasts. (**a**) HTFs were deprived of serum for 24 h, incubated in the absence or presence of BAC (5 × 10^−6^%) for 24 h, and then subjected to immunofluorescence staining with antibodies to αSMA (green). Nuclei were counterstained with 4',6-diamidino-2-phenylindole (DAPI, blue). Scale bars, 100 µm. (**b**) Cells treated as in (**a**) were subjected to immunoblot analysis with antibodies to αSMA and to α-tubulin (loading control). Blots for three independent experiments (Exp.) as well as quantitative data (means + s.e.m.) for the relative αSMA/α-tubulin band intensity ratio from four independent experiments are shown. *p < 0.05 (Student’s *t* test). Full-length blots are presented in Supplementary Fig. 1.

We next examined the effect of BAC at the nontoxic concentration on the phenotype of HTFs. Immunofluorescence analysis revealed that BAC induced marked expression of αSMA, a key marker of myofibroblast differentiation^9,10^, in these cells (Fig. 2a). Immunoblot analysis confirmed that BAC induced a significant increase in the abundance of αSMA in HTFs (2.02-fold, vs. BAC-untreated, *p* = 0.011, Fig. 2b).

### Effect of HCE cells on the BAC-induced transdifferentiation of HTFs into myofibroblasts

**Figure 3:**
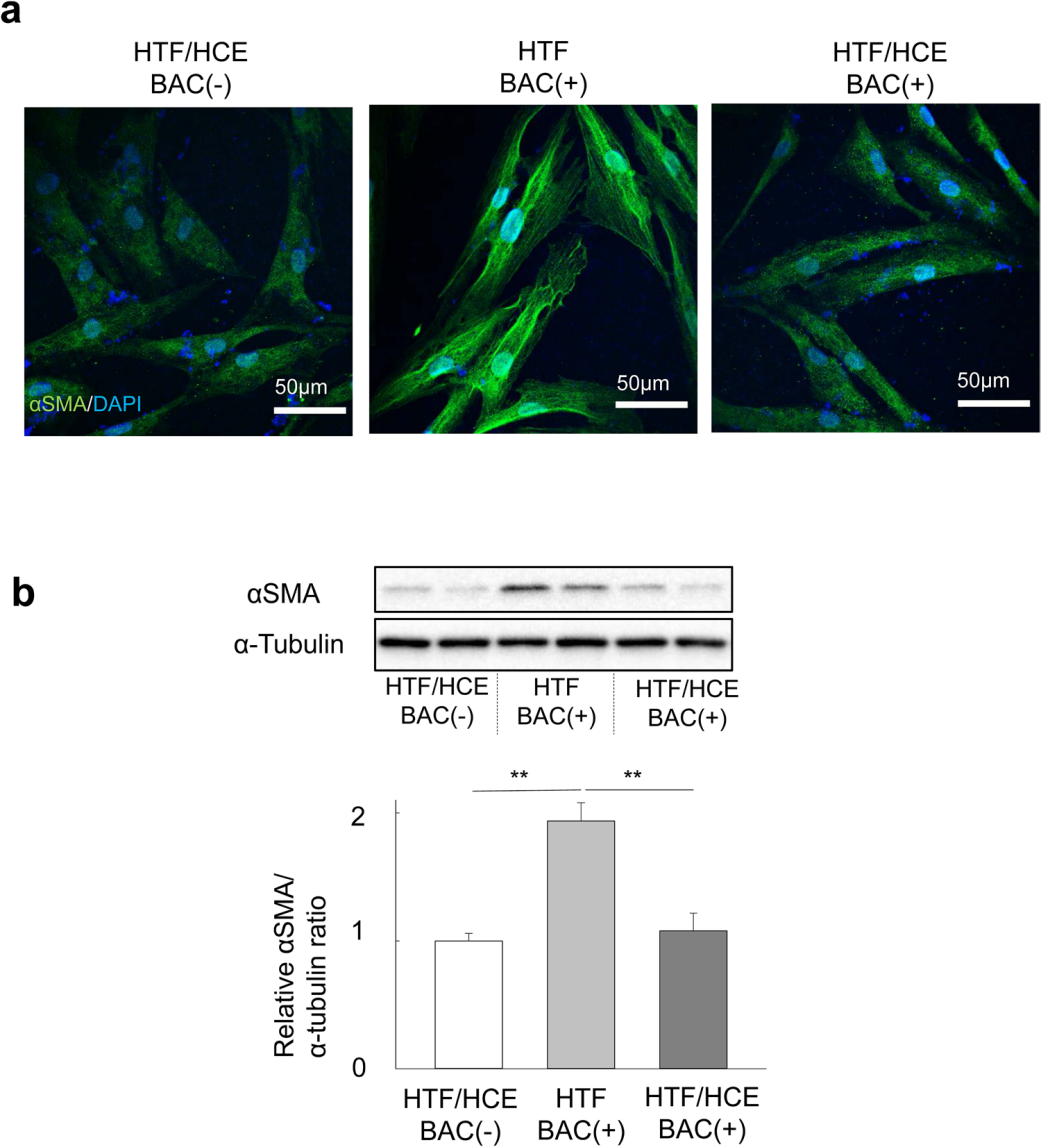
Effect of HCE cells on the BAC-induced transdifferentiation of HTFs into myofibroblasts in a coculture system. (**a**) HTFs cultured in hanging mesh inserts of six-well plates containing (or not) HCE cells were deprived of serum for 24 h, incubated in the absence or presence of BAC (5 × 10^−6^%) for 24 h, and then subjected to immunofluorescence staining with antibodies to αSMA (green). Nuclei were counterstained with DAPI (blue). Scale bars, 50 µm. (**b**) HTFs treated as in (**a**) were subjected to immunoblot analysis with antibodies to αSMA. Blots for duplicates of one representative experiment as well as quantitative data (means + s.e.m.) for the relative αSMA/α-tubulin band intensity ratio from five independent experiments are shown. **p < 0.01 (Holm-Sidak post hoc test). Full-length blots are presented in Supplementary Fig. 2.

Given that the corneal epithelium and subconjunctival tissue including Tenon’s capsule fibroblasts interact with each other through tear fluid^19^, we examined the effect of HCE cells on the BAC-induced myofibroblastic transdifferentiation of HTFs in a coculture system. HCE cells were thus cultured on the bottom of six-well plates, and HTFs were cultured in hanging mesh inserts. The cells were deprived of serum for 24 h and then exposed to BAC for 24 h. Immunofluorescence analysis revealed that HTFs cultured in the inserts in the absence of HCE cells manifested BAC-induced expression of αSMA, whereas this effect was greatly suppressed in HTFs cocultured with HCE cells (Fig. 3a). Again, these results were confirmed by immunoblot analysis (Fig. 3b).

### Effects of BAC on cytokine release in the HTF/HCE cell coculture system

**Figure 4:**
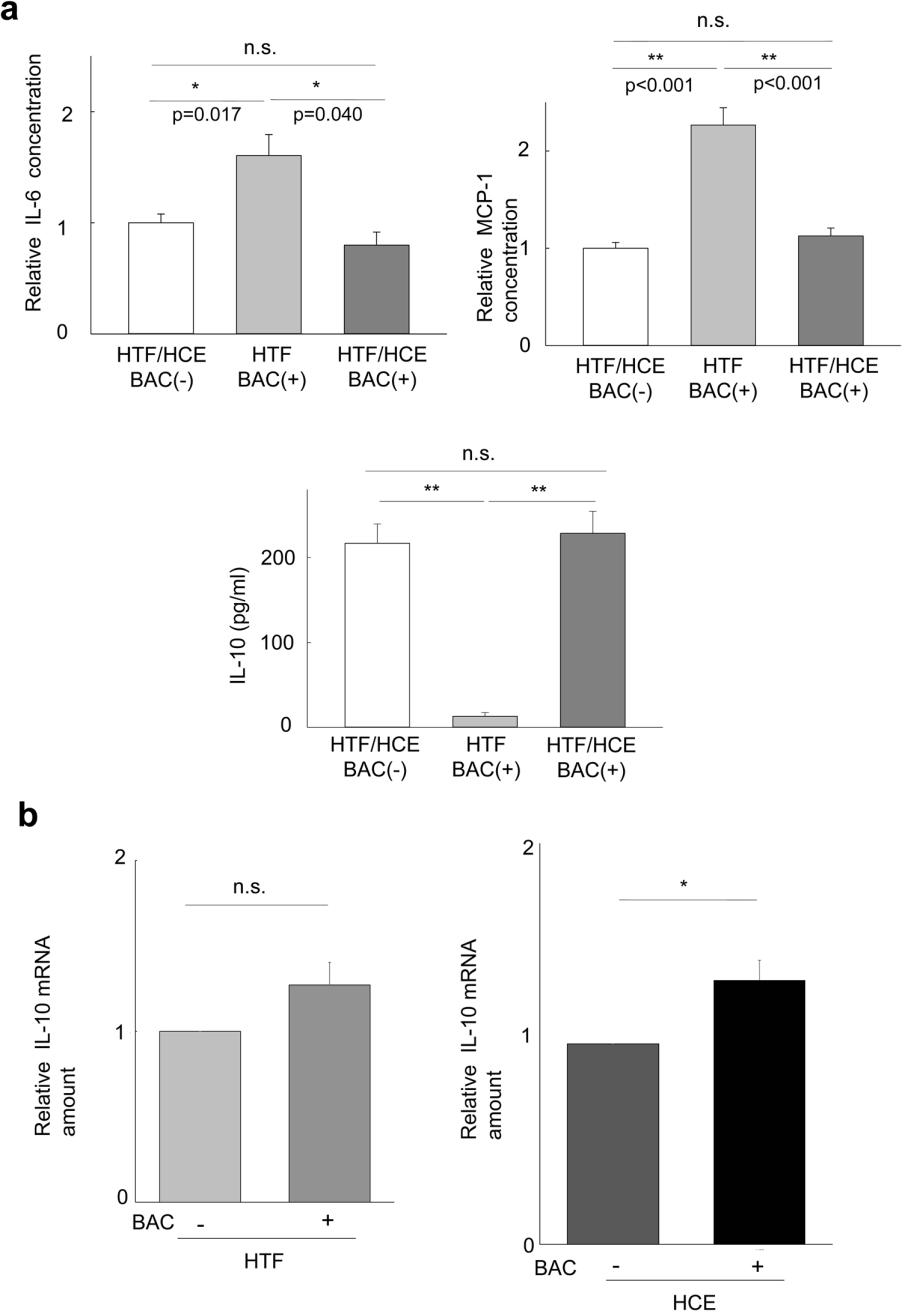
Effects of BAC on cytokine release in an HTF/HCE cell coculture system. (**a**) HTFs cultured in hanging mesh inserts of six-well plates containing (or not) HCE cells were deprived of serum for 24 h and then incubated in the absence or presence of BAC (5 × 10^−6^%) for 24 h, after which the levels of IL-6, MCP-1, and IL-10 in culture supernatants of the HTFs were measured. Data are means + s.e.m. for four independent experiments. *p < 0.05, **p < 0.01, n.s. (Holm-Sidak post hoc test). (**b**) HTFs and HCE cells were cocultured and treated as in (**a**), after which the abundance of IL-10 mRNA in the two cell types was determined by RT-qPCR analysis. Data were normalized by the amount of GAPDH mRNA, and the normalized values were expressed relative to that for the corresponding cells incubated without BAC. Data are means + s.e.m. for three independent experiments. *p < 0.05, n.s. (Student’s *t* test).

To explore the mechanism by which HCE cells might suppress BAC-induced expression of αSMA in HTFs, we measured the concentrations of IL-6, monocyte chemoattractant protein-1 (MCP-1), and IL-10 released into the culture medium. The levels of IL-6 and MCP-1 in culture supernatants of BAC-treated HTFs cultured alone were significantly greater than those for BAC-treated cocultures of HTFs and HCE cells (*p* < 0.01, Fig. 4a). The levels of IL-6 and MCP-1 did not differ significantly between HTF/HCE cell cocultures maintained with or without BAC. In contrast, the concentration of IL-10 in the medium of BAC-treated HTFs cultured alone was only 5.79% of that for BAC-treated cocultures of HTFs and HCE cells (*p* < 0.001, Fig. 4a). Again, the level of IL-10 did not differ significantly between HTF/HCE cell cocultures maintained with or without BAC. To examine which of the two cell types might be responsible for the production of IL-10, we also determined the abundance of IL-10 mRNA by reverse transcription (RT) and quantitative polymerase chain reaction (qPCR) analysis in HTFs and HCE cells after coculture. Whereas the amount of IL-10 mRNA in HTFs did not differ significantly between cocultures maintained with or without BAC, that in HCE cells was significantly increased by BAC (1.32-fold, vs. HCE without BAC, *p* = 0.033, Fig. 4b).

### Effect of IL-10 on the BAC-induced transdifferentiation of HTFs into myofibroblasts

**Figure 5:**
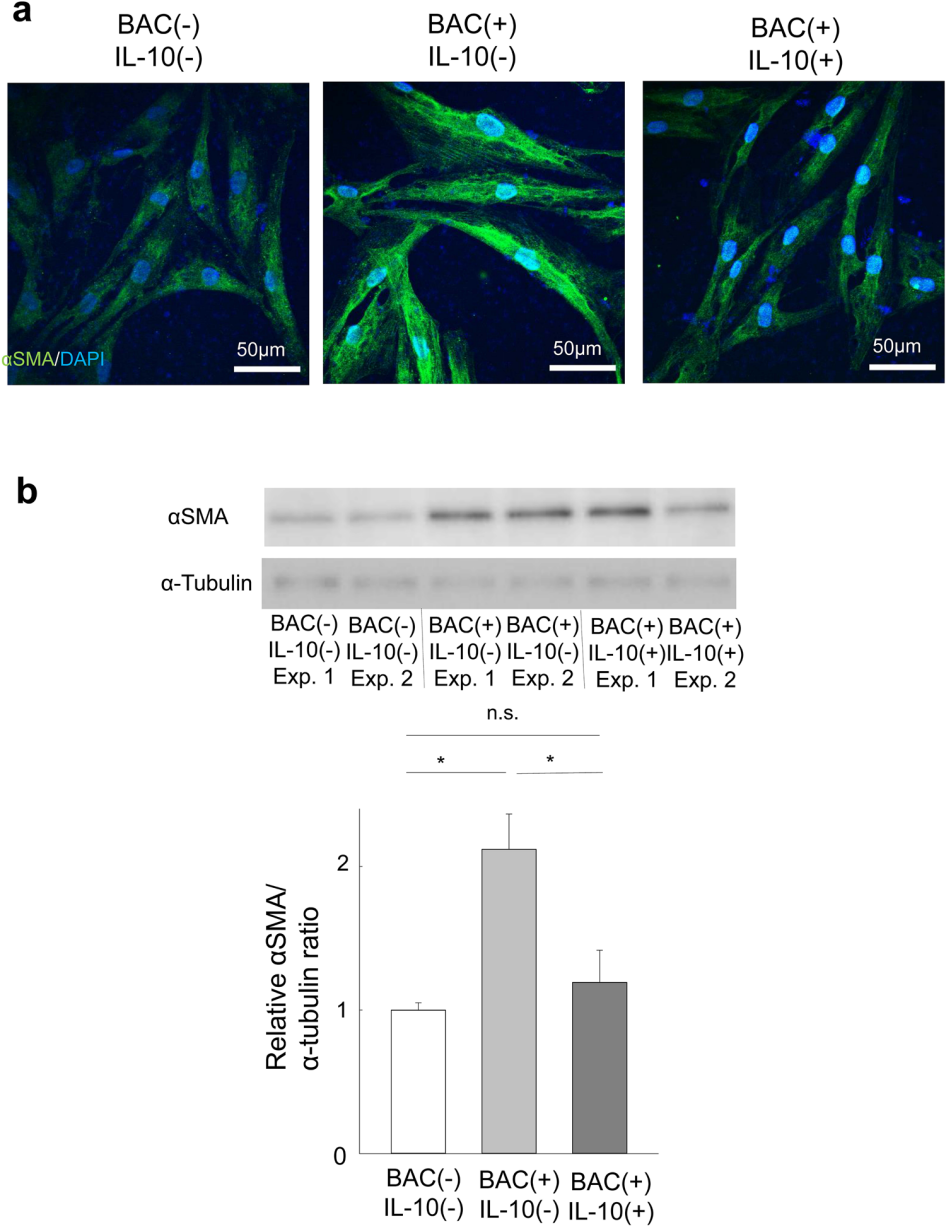
Effect of IL-10 on the BAC-induced transdifferentiation of HTFs into myofibroblasts. (**a**) HTFs were incubated first with or without IL-10 (300 pg/ml) for 24 h and then in the additional absence or presence of BAC (5 × 10^−6^%) for 24 h, after which the cells were subjected to immunofluorescence staining with antibodies to αSMA (green). Nuclei were counterstained with DAPI (blue). Scale bars, 50 µm. (**b**) Cells treated as in (**a**) were subjected to immunoblot analysis with antibodies to αSMA. Blots for two independent experiments as well as quantitative data (means + s.e.m.) for the relative αSMA/α-tubulin band intensity ratio from five independent experiments are shown. *p < 0.05, n.s. (Holm-Sidak post hoc test). Full-length blots are presented in Supplementary Fig. 3.

Given that the level of IL-10 in culture supernatants of HTF/HCE cell cocultures was increased compared with that for HTFs alone in the presence of BAC, we hypothesized that IL-10 might contribute to the attenuation by HCE cells of the BAC-induced transdifferentiation of HTFs into myofibroblasts. We therefore examined whether exogenous IL-10 might inhibit this effect of BAC. HTFs were incubated for 24 h with IL-10 (300 pg/ml) in serum-free medium and then exposed to BAC for an additional 24 h. Immunofluorescence staining (Fig. 5a) and immunoblot analysis (Fig. 5b) indeed showed that the BAC-induced expression of αSMA in HTFs was essentially abolished in the presence of IL-10.

### Effects of BAC and IL-10 on the subcellular localization of MRTF-A in HTFs

**Figure 6:**
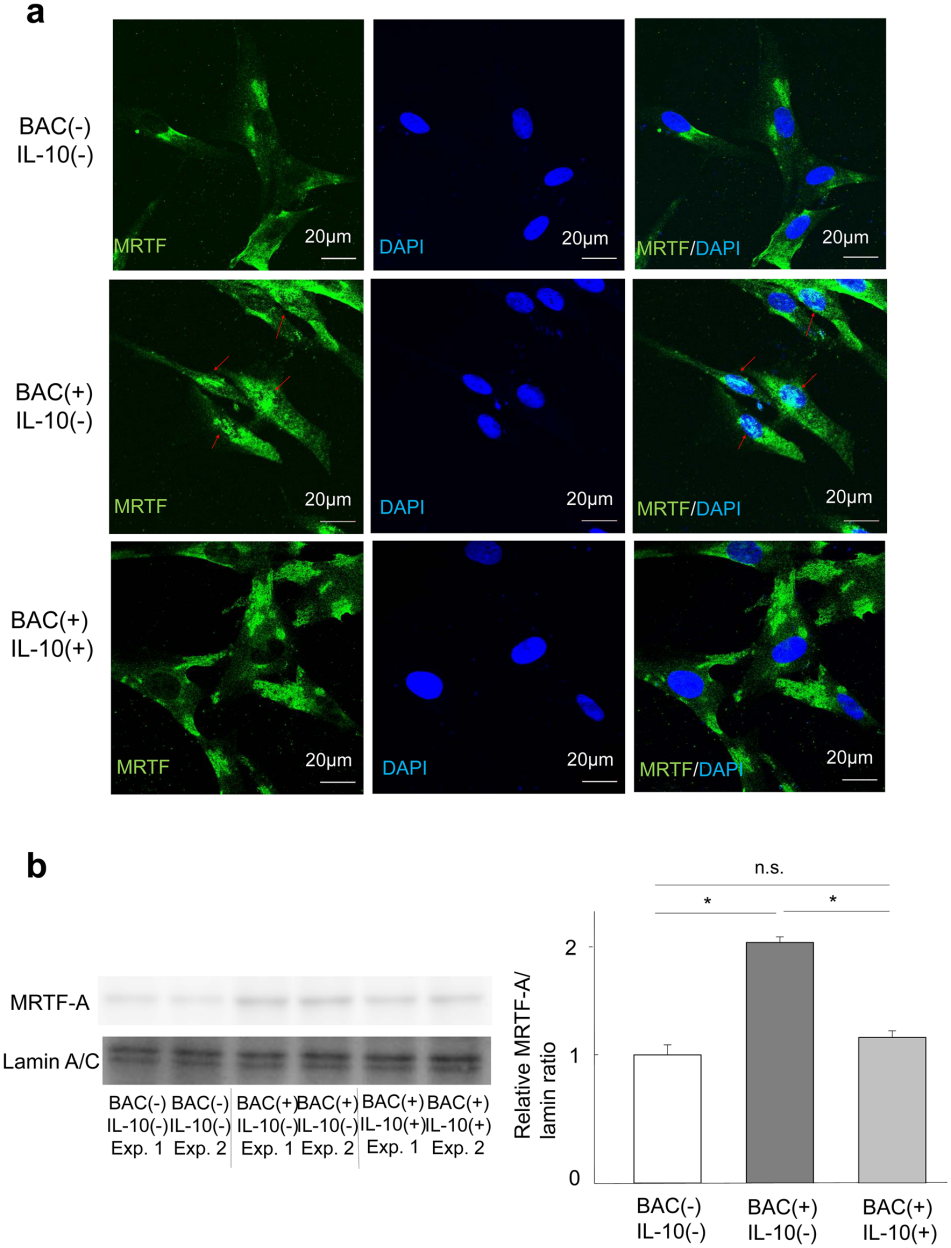
Effects of BAC and IL-10 on the subcellular localization of MRTF-A in HTFs. (**a**) HTFs were incubated first with or without IL-10 (300 pg/ml) for 24 h and then in the additional absence or presence of BAC (5 × 10^−6^%) for 6 h, after which the cells were subjected to immunofluorescence staining with antibodies to MRTF-A (green). Nuclei were counterstained with DAPI (blue). Arrows indicate localization of MRTF-A to the nucleus. Scale bars, 20 µm. (**b**) Cells treated as in (**a**) with the exception that they were exposed (or not) to BAC for 24 h were subjected to subcellular fractionation, and the nuclear fraction was subjected to immunoblot analysis with antibodies to MRTF-A and to lamin A/C (nuclear marker). Blots for two independent experiments as well as quantitative data (means + s.e.m.) for the relative αSMA/lamin band intensity ratio from four independent experiments are shown. *p < 0.05, n.s. (Holm-Sidak post hoc test). Full-length blots are presented in Supplementary Fig. 4.

We previously showed that EMT is mediated through MRTF-A signaling in retinal pigment epithelial cells^13^. We therefore investigated whether MRTF-A signaling might also play a role in the BAC-induced transdifferentiation of HTFs into myofibroblasts. Immunofluorescence analysis revealed that MRTF-A was localized predominantly to the cytoplasm of HTFs in the absence of BAC, whereas it had translocated to the nucleus in cells exposed to BAC (Fig. 6a). Importantly, this effect of BAC on MRTF-A localization was inhibited IL-10. The BAC-induced translocation of MRTF-A to the nucleus and its significant attenuation by 39.32% in cells exposed to IL-10 were confirmed by immunoblot analysis (*p* < 0.001, Fig. 6b).

## Discussion

Fibroblasts transdifferentiate into myofibroblasts in response to various stimuli. Myofibroblasts mediate ECM secretion, accumulation, and contraction and thereby contribute to fibrosis^9,20^. Activation of HTFs also initiates myofibroblast transdifferentiation and mediates subconjunctival fibrosis^9,16,17,21^. BAC was previously shown to induce conjunctival and subconjunctival fibrosis^1,4,22,23^. Furthermore, αSMA expression was found to be increased in the filtration surgery flap of a rabbit model of dry eye treated with BAC^18^. We have now shown that BAC increased the expression of αSMA in primary HTFs, suggesting that BAC triggers the transdifferentiation of HTFs into myofibroblasts and might thereby promote subconjunctival fibrosis.

BAC has toxic effects on the ocular surface and can be allergenic^1^. It induces ocular surface disorders including tear film instability by eliciting the loss of corneal epithelial cells, conjunctival cells, and subconjunctival cells^4,24^. The concentration of BAC as a preservative in eyedrops ranges from 0.004 to 0.02%^25,26^. We found that BAC induced the transdifferentiation of HTFs into myofibroblasts at a concentration of 5 × 10^−6^%, a concentration much lower than that present in eyedrops.

Components of the ocular surface include the cornea, conjunctiva, lacrimal glands, and eyelids, and they are interconnected functionally^27^. The corneal epithelium acts as a physical barrier and plays an important role in the ocular immune response^19^. In the present study, activation of HTFs by exposure to BAC was suppressed by the additional presence of HCE cells in a coculture system, indicating that corneal epithelial cells and HTFs may interact with each other in vivo and that such interaction contributes to homeostasis of the ocular surface.

Nuclear translocation of MRTF-A mediates activation of the expression of genes related to the myofibroblastic phenotype and fibrosis^9,11,12^. The MRTF-A pathway thus promotes αSMA expression in and scar tissue formation by conjunctival fibroblasts^28,29^. We have also previously shown that MRTF-A activation contributes to subretinal fibrosis^13^. MRTF-A forms a complex with G-actin and is located predominantly in the cytoplasm of cells under resting conditions^30,31^. Cell stimulation results in the dissociation of MRTF-A from G-actin and its transient translocation to the nucleus, where it interacts with serum response factor (SRF) and induces the expression of genes including that for αSMA^30,31^. The nuclear translocation of MRTF-A thus shifts the balance in the distribution of MRTF-A between the cytoplasm and the nucleus^32^. Our immunostaining and immunoblot data together showed that MRTF-A was essentially restricted to the cytoplasm, being absent from the nucleus, in HTFs under the control condition, whereas a proportion of the protein translocated to the nucleus in response to BAC exposure. Our present data thus show that BAC induces the nuclear translocation of MRTF-A as well as the expression of αSMA in HTFs, suggesting that BAC activates the MRTF-A-αSMA axis in these cells and thereby induces their transdifferentiation into myofibroblasts.

The corneal epithelium and subconjunctival tissue are connected to each other by tear fluid and the various factors that it contains^19^. Our results now indicate that the levels of IL-6 and MCP-1 in the culture supernatant of HTFs were increased by exposure to BAC, and that the BAC-induced increases in the concentrations of these inflammatory cytokines were significantly inhibited by coculture of HTFs with HCE cells. In contrast, the production of IL-10 appeared to be decreased in HTF cultures exposed to BAC, and this effect was prevented by coculture with HCE cells. IL-10 is an anti-inflammatory cytokine and maintains balance of the immune response^33^. It also inhibits the proliferation of and collagen synthesis by dermal, lung, and kidney cells^10^. Various studies have examined the possible roles of cytokines in ocular surface tissue fibrosis. The levels of IL-10 and IL-6 in tear fluid were found to be increased in individuals subjected to long-term treatment with eyedrops containing BAC^3^, suggesting a possible role for IL-10 in BAC-induced reactions at the ocular surface. Stevens-Johnson syndrome (SJS) and ocular cicatricial pemphigoid (OCP) are rare but severe autoimmune diseases that are associated with chronic inflammation of the ocular surface, stromal scarring, and symblepharon. Pseudo-OCP is induced by long-term use of topical ocular agents, especially that of antiglaucoma eyedrops containing preservatives. The conjunctiva of individuals with OCP was found to contain αSMA-expressing fibroblasts^34^ or to manifest overexpression of inflammatory cytokines^35^. The level of IL-10 in tear fluid was also shown to be decreased in patients with SJS compared with healthy individuals^34^. Subconjunctival injection of mesenchymal stem cells (MSCs) reduced the incidence of corneal allograft rejection in rats, and the abundance of IL-10 mRNA in the allografts was significantly higher in animals treated with MSCs than in those not so treated^36^. Furthermore, subconjunctival injection of IL-10-overexpresing bone marrow-derived MSCs into rat corneal allografts prolonged allograft survival^37^.

There is evidence that conjunctival cells produces intracellular cytokines including TNF-α and IL-6^3^, which are involved in transdifferentiation of human Tenon’s fibroblasts to myofibroblasts^38^, and excrete them in tears^3^. Their expression was also up-regulated in conjunctival scarring specimens obtained from patients with ocular surface inflammatory diseases^39,40^. In contrast, corneal epithelial cells secrete anti-inflammatory cytokines including IL-10 by BAC^41^. The relation between corneal epithelial cells and subconjunctival fibrosis has not previously been examined in vitro or in vivo. Our present results implicate cytokines in maintenance of homeostasis of ocular surface tissues exposed to BAC. The amount of IL-10 mRNA in HCE cells was significantly increased by exposure to BAC, suggesting that corneal epithelial cells may increase their production of IL-10 in response to treatment with eyedrops containing BAC. The amount of IL-10 mRNA in HTFs cocultured with HCE cells was also not decreased by exposure to BAC, suggesting that HCE cells might protect HTFs not only by producing IL-10 themselves but also by attenuating inhibition of IL-10 secretion from HTFs. In addition, we found that exogenous IL-10 inhibited the BAC-induced release of IL-6 and MCP-1 by HTFs cultured alone (*p* < 0.01). The concentrations of IL-6 and MCP-1 in culture supernatant of HTFs were thus increased 1.718 ± 0.241-fold and 1.940 ± 0.187-fold (means ± s.e.m. from three independent experiments), respectively, by exposure to BAC, whereas these values were reduced to 1.269 ± 0.101-fold and 1.560 ± 0.240-fold, respectively, in the additional presence of exogenous IL-10. These results thus indicate that IL-10 might ameliorate inflammatory and immune responses at the ocular surface including those in subconjunctival tissue. We further found that exogenous IL-10 inhibited the BAC-induced expression of αSMA and nuclear translocation of MRTF-A in HTFs, suggesting that IL-10 attenuates BAC-induced myofibroblastic transdifferentiation of HTFs by suppressing a BAC-MRTF-A-αSMA signaling pathway. IL-10 is therefore a potential therapeutic agent for tissue remodeling.

Our results suggest that corneal epithelial cells might play a key role in protection of Tenon fibroblasts from BAC-induced transdifferentiation into myofibroblasts. Furthermore, they implicate IL-10 as a key biological mediator in maintenance of homeostasis of ocular surface tissues, and they suggest that administration of IL-10 might protect Tenon fibroblasts from myofibroblastic transdifferentiation induced by BAC exposure. Our observations may provide the basis for a new strategy to prevent failure of glaucoma surgery due to BAC. The presence of drug-induced corneal epithelial damage might be a prognostic factor for glaucoma filtration surgery, with local application of IL-10 being a potential new approach to the control of subconjunctival fibrosis after such surgery. Moreover, IL-10 might also prove beneficial for topical ocular treatment in individuals with SJS or with OCP or pseudo-OCP.

In summary, we have examined for the first time the interaction of HTFs and HCE cells in a coculture system. We found that HCE cells inhibited the BAC-induced transdifferentiation of HTFs into myofibroblasts, and that this effect of HCE cells was mimicked by IL-10. Although the precise mechanism of this interaction between the two cell types is unclear, it may be mediated at the level of IL-10 production, given that the concentration of IL-10 in culture supernatants of HTFs was increased in the presence of HCE cells. BAC also induced the nuclear translocation of MRTF-A in HTFs, and this effect was again inhibited by IL-10, suggesting that MRTF-A mediates the effect of BAC on HTF transdifferentiation. Further studies are warranted to identify proteins that act upstream of MRTF-A in this pathway as well as to examine whether IL-10 might inhibit BAC-induced subconjunctival fibrosis in vivo.

## Methods

### Materials

Minimum essential medium (MEM), Dulbecco’s modified Eagle’s medium-nutrient mixture F12 (DMEM-F12), fetal bovine serum (FBS), gentamicin, and antibiotic–antimycotic mixture (15240-062) were obtained from Invitrogen-Gibco (Rockville, MD, USA). Cell culture dishes and six-well culture plates were from Corning (Corning, NY, USA), and Millicell Cell Culture Inserts were from Merck KGaA (Darmstadt, Germany). Bovine serum albumin (BSA), cholera toxin, bovine insulin, and mouse monoclonal antibodies to αSMA (F3777) and to α-tubulin (T5168) were obtained from Sigma-Aldrich (St. Louis, MO, USA). Recombinant human epidermal growth factor was obtained from Corning, rabbit polyclonal antibodies to MRTF-A (Mkl1, ab113264) were from Abcam (Cambridge, UK), and goat polyclonal antibodies to lamin A/C were from Santa Cruz Biotechnology (Dallas, TX, USA). Alexa Fluor 488–conjugated goat antibodies to mouse or rabbit immunoglobulin G and 10% normal goat serum were from Invitrogen (Carlsbad, CA, USA), and horseradish peroxidase–conjugated secondary antibodies were from Jackson ImmunoResearch Laboratories (West Grove, PA, USA). BAC was from Nacalai Tesque (Kyoto, Japan), and recombinant human IL-10 was from Peprotech (Rocky Hill, NJ, USA). An RNeasy Mini Kit was from Qiagen (Venlo, the Netherlands), ReverTra Ace qPCR RT Master Mix was from Toyobo (Osaka, Japan), and SYBR Green reagents were from Life Technologies (Carlsbad, CA, USA).

### Cell culture

Given that primary human corneal epithelial cells have a short life span in culture, we used an SV40-immortalized human corneal epithelial cell line that retains the phenotypic morphology and function of the parent cells^42^ in this study. HCE cells were obtained from RIKEN Biosource Center (Tsukuba, Japan) and were maintained under a humidified atmosphere of 5% CO_2_ and 95% air at 37 °C in DMEM-F12 supplemented with 10% FBS, insulin (5 μg/ml), cholera toxin (0.1 μg/ml), epidermal growth factor (10 ng/ml), and gentamicin (40 μg/ml)^43^. HTFs were isolated as described previously^44^ from individuals undergoing strabismus surgery who had no history of conjunctival disease or use of topical ocular medication. The use of these cells complied with the tenets of the Declaration of Helsinki and was approved by the human experimentation committee of Yamaguchi University Graduate School of Medicine, and informed consent was obtained from each donor. HTFs were maintained under an atmosphere of 95% air and 5% CO_2_ at 37 °C in MEM supplemented with 10% FBS and antibiotic–antimycotic mixture, and they were used for the present study after three to eight passages. For coculture of HTFs and HCE cells, HTFs cultured in hanging mesh inserts to 70–80% confluence were transferred to six-well plates containing HCE cells also at 70–80% confluence, and both cell types were maintained in the same medium (MEM).

### Assay of LDH

LDH released into culture supernatants was measured with a colorimetric assay (Cytotoxicity LDH Assay Kit-WST; Dojindo Laboratories, Kumamoto, Japan). The absorbance of samples was measured at 490 nm with a microplate reader (PowerWave XS; BioTek Instruments, Winooski, VT, USA).

### Immunofluorescence analysis

Cells cultured on glass coverslips were fixed with 4% paraformaldehyde for 30 min at room temperature, washed with phosphate-buffered saline (PBS), and permeabilized with 0.1% Triton X-100 in PBS for 15 min at room temperature. Cells cultured in hanging mesh inserts were fixed with methanol for 15 min at − 30 °C. All the samples were incubated overnight at 4 °C with 1% goat serum in PBS to block nonspecific binding. For staining of αSMA, cells were incubated first overnight at 4 °C with antibodies to αSMA (1:500 dilution in blocking solution) and then for 1 h at room temperature with Alexa Fluor 488-conjugated secondary antibodies (1:500 in blocking solution). For staining of MRTF-A, cells were incubated first overnight at 4 °C with antibodies to MRTF-A (1:150 in blocking solution) and then for 1 h at room temperature with Alexa Fluor 488-conjugated secondary antibodies (1:500 in blocking solution). Nuclei were stained with DAPI. Confocal images of cells on glass were obtained with a fluorescence microscope (BZ-X710; Keyence, Osaka, Japan), whereas those of cells in hanging inserts were obtained with a laser confocal microscope (Zeiss LSM510META).

### Immunoblot analysis

Cells were homogenized on ice in a lysis buffer (50 mM Tris–HCl [pH 7.5], 165 mM NaCl, 10 mM NaF, 1 mM sodium vanadate, 1 mM phenylmethylsulfonyl fluoride, 10 mM EDTA, aprotinin [10 μg/ml], leupeptin [10 μg/ml], 1% NP-40), the homogenate was centrifuged at 15,000 × *g* for 30 min at 4 °C, and the resulting supernatant was collected for immunoblot analysis. The samples (5 µg of protein) were fractionated by SDS–polyacrylamide gel electrophoresis on a 5–20% gradient gel (SuperSep Ace; Wako, Osaka, Japan) at a constant current of 20 mA per gel, after which the separated proteins were transferred to an Immobilon-P membrane (Millipore, Billerica, MA, USA). The membrane was exposed to 3% BSA in Tris-buffered saline (TBS) for 1 h at room temperature, washed with TBS containing 0.1% Tween-20, and incubated overnight at 4 °C with antibodies to αSMA (1:1000 dilution in TBS containing 3% BSA) or to α-tubulin (1:1000). For immunoblot analysis of nuclear fractions, antibodies to MRTF-A (1:1000) and to lamin A/C (1:2000) were used. The membrane was then incubated for 1 h at room temperature with horseradish peroxidase–conjugated secondary antibodies (1:10,000). The membrane was finally washed with PBS before detection of immune complexes with the use of ImmunoStar LD or ImmunoStar Zeta (Wako) reagents and a ChemiDoc instrument (Bio-Rad, Hercules, CA, USA). Band intensity was measured with Image Lab software (Bio-Rad).

### Assay of cytokines

The concentrations of cytokines (IL-6, D6050; IL-10, D1000B; MCP-1; DCP00) in the culture supernatants of HTFs cultured in hanging mesh inserts were measured with Quantikine ELISA kits (R&D Systems; Minneapolis, MN, USA). The absorbance of each well was determined with a microplate reader (PowerWave XS, BioTek Instruments) set to 450 nm, with the wavelength correction set to 540 nm.

### RT-qPCR analysis

Total RNA was isolated from cells in six-well plates or hanging mesh inserts with the use of an RNeasy Mini Kit and was subjected to RT with ReverTra Ace qPCR RT Master Mix. The obtained cDNA was subjected to qPCR analysis with the use of SYBR Green reagents and a StepOnePlus Real-Time PCR System (Applied Biosystems, Foster City, CA, USA). The qPCR primers (forward and reverse, respectively) were 5′-TCTCCGAGATGCCTTCAGCAGA-3′ and 5′-TCAGACAAGGCTTGGCAACCCA-3′ for IL-10 (NM_000572.3) and 5′-AGCCTCAAGATCATCAGCAAT-3′ and 5′-CCTTCCACGATACCAAAGTTGT-3′ for glyceraldehyde-3-phosphate dehydrogenase (GAPDH, NM_002046.5). The amount of IL-10 mRNA was normalized by that of GAPDH mRNA.

### Separation of nuclear and cytoplasmic proteins

Nuclear and cytoplasmic fractions were prepared from cell lysates with the use of NE-PER Nuclear and Cytoplasmic Extraction Reagents (Thermo Scientific, Waltham, MA, USA).

### Statistical analysis

Quantitative data are presented as mean + s.e.m. values and were analyzed with the two-tailed Student’s *t* test or by analysis of variance followed by the Holm-Sidak post hoc test for comparisons between two or among more than two groups, respectively. The analysis was performed with SigmaStat 13.0 software (Systat Software, San Jose, CA, USA). A *P* value of < 0.05 was considered statistically significant.

## Supplementary Information

Supplementary Information.

## Data Availability

The data sets generated and analyzed during the current study are available from the corresponding author on reasonable request.
