# Supplementary material for: Benzalkonium chloride-induced myofibroblastic transdifferentiation of Tenon’s capsule fibroblasts is inhibited by coculture with corneal epithelial cells or by interleukin-10
Source: Sci Rep. 2021 Aug 9;11:16096. doi: 10.1038/s41598-021-94852-8 (PMC8352883; doi:10.1038/s41598-021-94852-8)
Supplement: Supplementary file 1 — Supplementary Information. [file 41598_2021_94852_MOESM1_ESM.docx]

Supplementary Information

Benzalkonium chloride-induced myofibroblastic transdifferentiation of Tenon’s capsule fibroblasts is inhibited by coculture with corneal epithelial cells or by interleukin-10

Chiemi Yamashiro, Kazuhiro Tokuda, Yuka Kobayashi, Fumiaki Higashijima, Takuya Yoshimoto, Manami Ota, Tadahiko Ogata, Atsushige Ashimori, Masaaki Kobayashi, Makoto Hatano, Sho-Hei Uchi, Makiko Wakuta, Shinichiro Teranishi & Kazuhiro Kimura

Supplementary Information


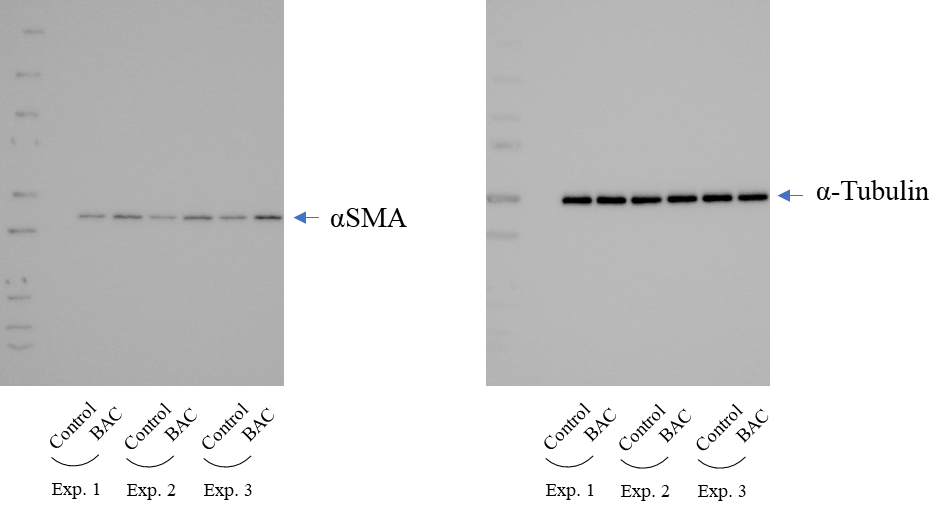


Figure S1. Full length images of Figure 2b


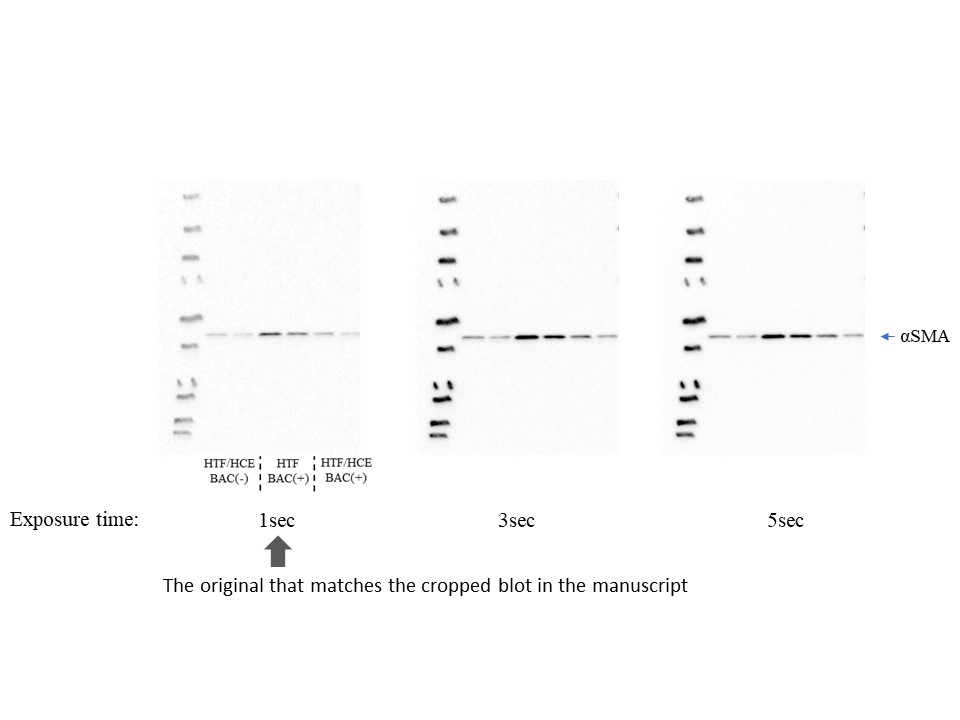
Figure S2-1. Full length images of Figure 3b (α-SMA) with multiple exposure time

Supplementary Information


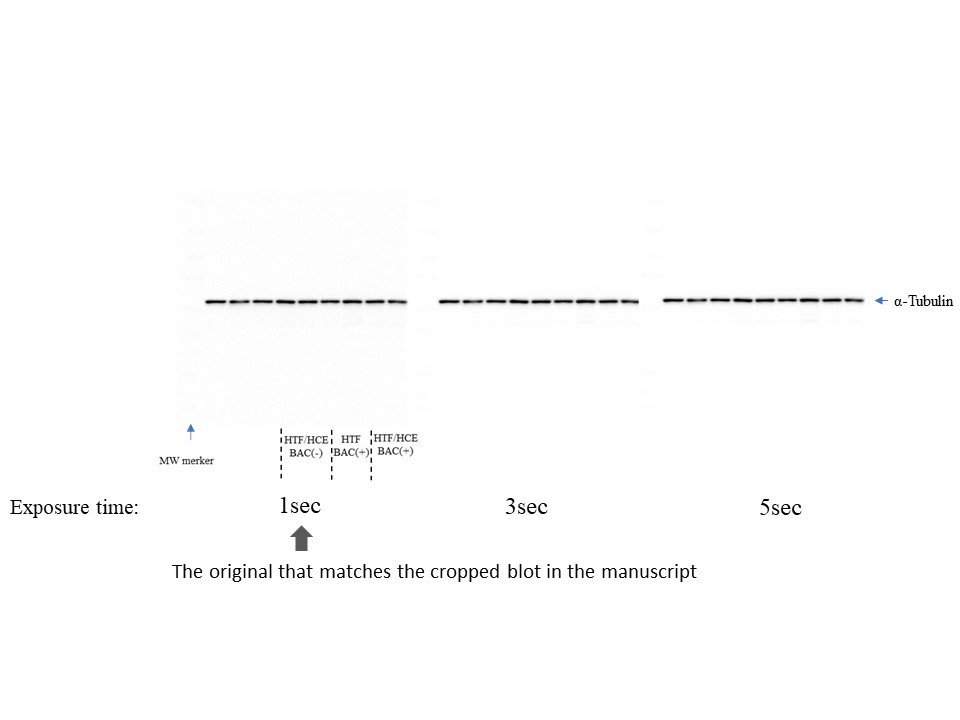


Figure S2-2. Full length images of Figure 3b (α-Tubulin) with multiple exposure time


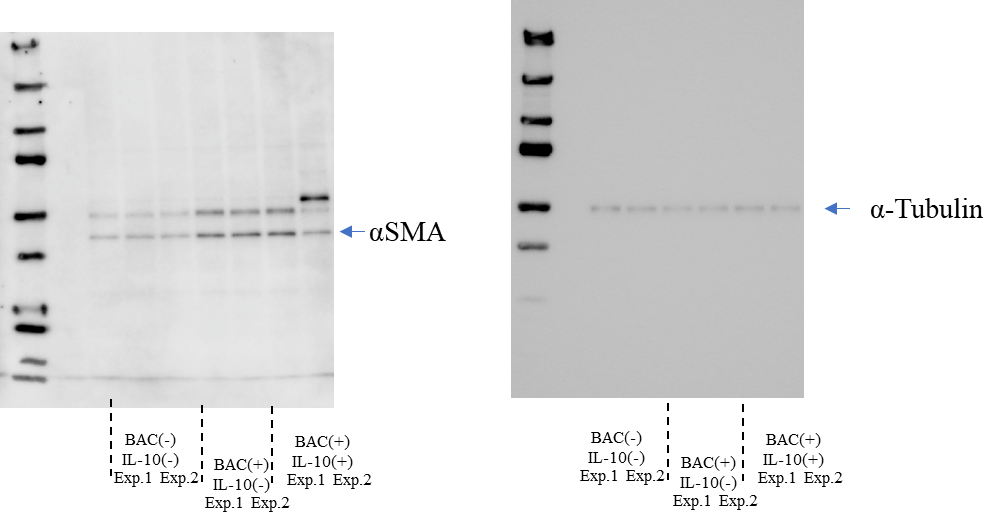
Figure S3. Full length images of Figure 5b

Supplementary Information


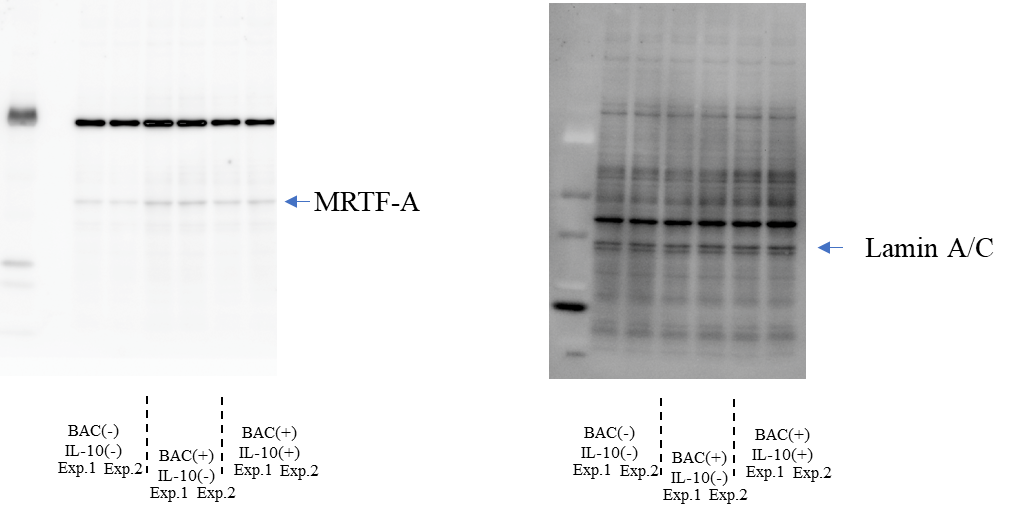


Figure S4. Full length images of Figure 6b
